# Blocking TGF**β** via Inhibition of the **α**v**β**6 Integrin: A Possible Therapy for Systemic Sclerosis Interstitial Lung Disease

**DOI:** 10.1155/2011/208219

**Published:** 2011-10-16

**Authors:** Tamiko R. Katsumoto, Shelia M. Violette, Dean Sheppard

**Affiliations:** ^1^Department of Medicine, Critical Care, Allergy and Sleep Medicine, University of California, San Francisco, CA 94143, USA; ^2^Stromedix, Cambridge, MA 02141, USA

## Abstract

Interstitial lung disease (ILD) is a commonly encountered complication of systemic sclerosis (SSc) and accounts for a significant proportion of SSc-associated morbidity and mortality. Its pathogenesis remains poorly understood, and therapies that treat SSc ILD are suboptimal, at best. SSc ILD pathogenesis may share some common mechanisms with other fibrotic lung diseases, in which dysregulation of lung epithelium can contribute to pathologic fibrosis via recruitment or in situ generation and activation of fibroblasts. TGF**β**, a master regulator of fibrosis, is tightly regulated in the lung by the integrin **α**v**β**6, which is expressed at low levels on healthy alveolar epithelial cells but is highly induced in the setting of lung injury or fibrosis. Here we discuss the biology of **α**v**β**6 and present this integrin as a potentially attractive target for inhibition in the setting of SSc ILD.

## 1. Introduction

Systemic sclerosis (SSc), also known as scleroderma, is a connective tissue disease of unknown etiology that is characterized by fibroproliferative changes in multiple organs, as well as microvascular and immunologic dysregulation. One of the most morbid conditions associated with SSc is interstitial lung disease (ILD), which occurs in 25–90% of SSc patients, depending on the detection methods used and the demographics of the population being studied [1, 2]. The pathologic mechanisms responsible for the initiation and maintenance of SSc ILD remain poorly characterized. Approximately 42% of patients with SSc ILD will die of disease progression within 10 years of diagnosis [3], and currently no curative therapies exist to combat this morbid complication.

## 2. Mechanisms of Fibrosis in SSc: A Role for Epithelial Cells?

Much of the research literature on SSc-associated fibrosis has focused on the roles of fibroblasts and myofibroblasts, the effector cells that are ultimately involved in the production of collagen and other extracellular matrix (ECM) proteins. However, the development of fibrosis in SSc is indeed a complex process involving crosstalk amongst multiple cell types, including epithelial, endothelial, immune, and mesenchymal cell types. In idiopathic pulmonary fibrosis (IPF), a progressive fibrosing lung disease that has a median survival of between two and three years [4], the principle defect is thought to be recurrent epithelial injury with resultant epithelial cell senescence and/or apoptosis. Epithelial injury can lead to the recruitment and activation of fibroblasts, which can be derived from resident fibroblasts, circulating fibrocytes, or the differentiation of epithelial cells, endothelial cells, or pericytes into fibroblasts. The best characterized of these changes in cell differentiation involves epithelial cells and has been termed epithelial-to-mesenchymal transition (EMT). Alveolar type II epithelial cell (AT2) injury has long been observed in lung biopsies from patients with ILD, and recent animal data suggests a causal relationship between AT2 injury and fibrosis. Sisson et al. recently demonstrated that targeted deletion of AT2 cells, using diphtheria toxin driven by a specific lung epithelial cell promoter leads directly to lung fibrosis [5]. The most convincing evidence for the contribution of EMT to lung fibrosis came from studies by Kim et al., who used genetic fate-mapping methods to demonstrate the capacity of alveolar epithelial cells to undergo EMT in an established mouse model of lung fibrosis [6]. Based on these data and others, injured alveolar epithelial cells are viewed as potential drivers of pathologic pulmonary fibrosis.

Prior studies have provided evidence for increased epithelial cell damage in SSc ILD. Wells et al. measured the speed of clearance of technetium-labeled diethylene-triamine-pentaacetate (^99m^Tc-DTPA) from the lungs in 53 patients with SSc ILD and found that rapid clearance, which suggested breach of epithelial barrier function, was associated with more dramatic clinical deterioration whereas normal clearance predicted stable disease [7]. Serum levels of the mucin-like glycoprotein KL-6, which is produced exclusively by lung epithelial cells and is associated with lung epithelial cell damage, are increased in ILD associated with connective tissue diseases [8]. 

Recurrent lung epithelial injury via chronic microaspiration has been proposed as a mechanism contributing to lung fibrosis. After the skin, the most commonly affected organ system in SSc is the gastrointestinal tract, affecting approximately 50–90% of all patients [9–11]. The esophagus is the most frequently involved site of the GI tract, leading to gastroesophageal reflux (GER). In a rodent model, chronic gastric fluid aspiration leads to a lymphocytic and obliterative bronchiolitis as well as parenchymal fibrosis, with increased TGF*β* levels in bronchoalveolar lavage fluid [12]. Intriguingly, these histologic changes are independent of gastric fluid pH. The bile acid chenodeoxycholic acid stimulates TGF*β* production in human airway epithelial cells and induces fibroblast proliferation *in vitro* in a TGF*β*-dependent manner [13]. Correlative data support a relationship between chronic microaspiration and SSc ILD as well as other fibrotic lung diseases such as IPF [14, 15]. A strong association between GER and IPF has been recently reported in several studies, with an estimated prevalence of 67–88% for distal esophageal reflux and 30–71% for proximal esophageal reflux based on 24-hour esophageal pH monitoring. Interestingly, symptoms of reflux were poor predictors for the diagnosis of GER, implying a significant component of silent microaspiration [16–18]. Besides microaspiration, other mechanisms leading to lung fibrosis could also be at play in SSc ILD, involving not only epithelial cells but also endothelial, mesenchymal, and immune cell types. However, the hypothesis that microaspiration leads to SSc pulmonary fibrosis via recurrent epithelial injury is certainly an important one that needs to be strongly considered, especially given the prevalence of GER in SSc.

## 3. TGF***β***: A Critical Mediator of Fibrosis

TGF*β* is a pleiotropic cytokine that affects cell proliferation, differentiation, and apoptosis and is involved in a multitude of homeostatic functions. Importantly, TGF*β* is regarded as the “master switch” of fibrosis in many tissues, including the lung [19]. The major effects of TGF*β* include inhibition of epithelial cell proliferation, induction of fibroblast proliferation and the expression of genes encoding components of the ECM, and inhibition of the expression of metalloproteinase genes. TGF*β* can stimulate fibroblast conversion into contractile myofibroblasts, which actively produce collagen and other ECM proteins, and may serve as an inducer of EMT, leading to fibrosis [20]. Mice that possess a gain of function mutation in the TGF*β* pathway develop progressive fibrosis in multiple organs resembling SSc [21]. Global deletion of Smad3, a critical mediator of TGF*β* signaling, or specific deletion of the TGF*β* receptor II from lung epithelial cells affords resistance to bleomycin-induced lung fibrosis [22, 23]. 

Much data underscores the importance of TGF*β* in SSc-associated fibrosis [24]. Increased expression of TGF*β*1 or TGF*β*2 is seen in early skin lesions and in lung tissue from patients with SSc ILD [25, 26], and TGF*β*1 was significantly elevated in bronchoalveolar lavage fluid from SSc patients with pulmonary fibrosis [27]. A critical role for TGF*β* in SSc has been highlighted by DNA microarray studies of SSc skin and fibroblasts. Recently, Sargent et al. generated a TGF*β*-responsive signature in dermal fibroblasts comprised of 894 responsive genes [28]. Analysis of these genes in SSc skin biopsies revealed that this TGF*β*-responsive signature occurred exclusively in a subset of skin biopsies from patients with diffuse SSc, and in particular, those who had a higher incidence of lung fibrosis. Importantly, these data suggest that a subset of SSc patients has disease that is predominantly driven by TGF*β*.

## 4. Regulation of TGF***β*** by ***α***v***β***6

There are three isoforms of TGF*β* in mammals which are all bind to the same heteromeric receptor, leading to activation of the canonical pathway via phosphorylation of Smad proteins. In addition, noncanonical pathways are activated by TGF*β* receptors, including several protein kinases (p38, JNK, Erk, c-Abl, TGF-*β*-activated kinase) and the lipid kinase PI3 kinase and its downstream target Akt. However, the phenotypes of mice lacking the different TGF*β* isoforms are disparate, which could be explained by differences in isoform expression patterns or differential regulation of non-canonical signaling pathways. 

Mice deficient in TGF*β*1 exhibit uncontrolled tissue inflammation, autoimmunity, and premature death, demonstrating a critical role for TGF*β*1 in immune homeostasis [29, 30]. These data suggest that general blockade of TGF*β* should be approached with caution. A clinical trial of SSc patients utilizing an antibody directed against TGF*β*1 showed no appreciable therapeutic effect [31], although the potency of this antibody has been questioned. Given its pleiotropic effects, TGF*β* inhibition using strategies targeted to specific regions involved in fibrosis might be a better alternative. Most other approaches currently under consideration for targeting TGF*β* block either TGF*β* receptors or TGF*β* itself. These approaches might lead to unwanted side effects by interfering with important homeostatic effects of TGF*β* at sites outside the organs affected by tissue fibrosis. Although mice lacking *α*v*β*6 do have mild inflammation in the lungs and skin, these effects are much less severe than those seen in mice lacking even a single TGF*β* isoform. Additionally, the *α*v*β*6 integrin is highly upregulated in diseased tissue providing a mechanism for injury-induced TGF*β* activation as compared to homeostatic control of TGF*β* activity. By inhibiting only a subset of TGF*β* activation, particularly in injured epithelial organs, targeting *α*v*β*6 could allow treatment of tissue fibrosis with substantially reduced risk of disrupting beneficial homeostatic control of inflammation and immunity.

The regulation of TGF*β* activity involves multiple interactions of various proteins with the TGF*β* cytokine. TGF*β* is normally secreted as a complex which includes the bioactive peptide of TGF*β*1, an amino terminal fragment of the TGF*β*1 gene product called the latency-associated peptide (LAP), and the latent TGF*β*-binding protein (LTBP) [32]. The TGF*β* gene product is cleaved within the endoplasmic reticulum by the endopeptidase, furin, and it is assembled as a complex of two disulfide-linked homodimers formed from the shorter carboxy-terminal fragment (the active cytokine) and the longer amino-terminal fragment, LAP. These two homodimers associate noncovalently to form the small latent complex, which is unable to activate the TGF*β* receptor because LAP shields the mature TGF*β* homodimer from interaction with its receptor. In most cells, this small latent complex becomes disulfide linked to one of the latent TGF*β*-binding proteins (LTBP). This large complex is secreted and attaches to components of the extracellular matrix and is covalently cross-linked to ECM proteins via the action of extracellular tissue transglutaminase. This preformed latent TGF*β* complex exists at a high concentration in the ECM of most organs with little evidence of TGF*β* activation [33]. Given the diverse and potent effects of TGF*β*, its activity must be tightly regulated in a spatially specific manner.

Integrins are cell surface molecules comprised of alpha and beta chain heterodimers that regulate cell adhesion, survival, proliferation, and migration [34]. Pulmonary epithelial cells express at least 8 distinct integrin heterodimers. The *α*3*β*1 and *α*6*β*4 integrins recognize the epithelial basement protein, laminin 5 and play an important role in maintenance of epithelial integrity [35–38]. The other 6-lung epithelial integrins recognize ligands that are not present at baseline but are components of the provisional matrix that are upregulated in response to injury or inflammatory stimuli. The *α*v*β*6 integrin is the only integrin that is restricted in its expression to epithelial cells. This integrin, minimally expressed in healthy airway and alveolar epithelial cells at baseline, gets rapidly induced at these sites in response to a variety of insults, including lung injury [39]. Notably, and of possible relevance to the skin fibrosis of SSc, *α*v*β*6 is also upregulated on keratinocytes in the setting of wound healing but is minimally expressed at baseline [40]. *In vitro*, the *α*v*β*6 integrin binds to a number of ligands, including fibronectin, tenascin-C, and osteopontin [41] via interactions with an arginine-glycine-aspartic acid (RGD) tripeptide sequence, a sequence also recognized by several other integrins including those that share the *α*v subunit [42]. However, the *in vivo* relevance of *α*v*β*6 interactions with these ligands remains uncertain.

Mice completely lacking the *β*6 integrin subunit, which pairs exclusively with the *α*v subunit, were viable with a near-normal life expectancy, but developed low-grade inflammation of the skin and lungs and late-onset emphysema [43, 44]. Following intratracheal delivery of bleomycin, a drug used to induce pulmonary fibrosis, *β*6 deficient mice developed exaggerated inflammation in the lung but were remarkably protected from the subsequent development of pulmonary fibrosis [45]. These mice were also dramatically protected from radiation-induced pulmonary fibrosis [46]. These phenotypic findings suggested a role for the *α*v*β*6 integrin in regulating TGF*β*, a key negative regulator of inflammation but a positive regulator of fibrosis. Amino acid sequence analysis revealed the presence of an RGD-binding sequence in the latency-associated peptide (LAP) of TGF*β*1 and 3, and LAP*β*1 and *β*3 were demonstrated to be bona fide ligands for *α*v*β*6 [47, 48]. Cells expressing the *α*v*β*6 integrin were shown to generate TGF*β* activity that could be detected by an *in vitro* TGF*β* reporter assay, and this activity was dependent upon cell-cell contact and could be specifically blocked with antibodies to *α*v*β*6 [45]. Microarray analysis of lungs from mice treated with bleomycin revealed a large group of TGF*β*-inducible genes that were induced at much lower levels in the *β*6 knockout mice compared with wild-type mice [49]. Collectively, these data provide strong evidence that the *α*v*β*6 integrin on lung epithelial cells is an important regulator of TGF*β* activation. Activation could be inhibited by blocking actin polymerization [45] and by inhibitors of Rho kinase [50], suggesting a role for force generation by the actin cytoskeleton which presumably alters the conformation of latent complexes tethered to the extracellular matrix by matrix-bound LTBP, allowing for exposure of the active TGF*β* cytokine and its interaction with TGF*β* receptors. 

Regulation of TGF*β* activity in the lung was found to play an important role in the maintenance of alveolar homeostasis. Low-grade inflammation in the lungs of the *β*6 knockout mice was characterized by increased numbers of alveolar macrophages, neutrophils, lymphocytes, and eosinophils, and this inflammation was reversed by transgenic overexpression of constitutively active TGF*β* [44]. Microarray analysis of *β*6 deficient lungs showed more than a 20-fold increase in the expression of matrix metalloproteinase 12 (MMP12) [49]. This protease, which is predominantly expressed by macrophages, preferentially degrades elastin, and has been implicated in the pathogenesis of emphysema. Emphysema was noted in older *β*6 deficient mice, and crossing the *β*6 deficient mice with mice lacking MMP12 completely rescued this phenotype. Expression of a wild-type form of the *β*6 integrin prevented emphysema development, while expression of a mutant *β*6 integrin subunit unable to support TGF*β* activation did not prevent emphysema development. Studies have shown that the development of emphysema in *β*6 deficient mice correlates tightly with the upregulation of MMP12, suggesting that MMP12 could serve as a surrogate biomarker to assess for this particular consequence [44].

## 5. Rationale for ***α***v***β***6 Inhibition in SSc ILD

SSc ILD can be histopathologically classified as nonspecific interstitial pneumonia (NSIP) or usual interstitial pneumonia (UIP) [51–54]. NSIP is the more commonly encountered histopathologic subtype, comprised of varying degrees of inflammation and fibrosis, with some forms being predominantly inflammatory (cellular NSIP) and others primarily fibrotic (fibrotic NSIP). It remains unclear whether cellular NSIP and fibrotic NSIP represent a progression of one underlying disease process or rather two separate disease phenotypes, which in some cases can coexist within the same patient [55]. UIP is the pathologic pattern observed in idiopathic pulmonary fibrosis (IPF) and can also be seen in SSc ILD. UIP consists of interstitial fibrosis in a patchy pattern, honeycomb changes (both macroscopic and microscopic), and foci of fibroblastic proliferation. Although the UIP pattern in SSc is less commonly encountered clinically, it can be seen with increased frequency in patients with more severe fibrotic lung disease, including those with end-stage SSc ILD requiring lung transplant. 

Currently, there are no highly effective agents for the treatment of fibrotic lung diseases. Several studies using anti-TGF*β* agents have demonstrated protection from lung fibrosis in disease models [46, 56, 57]. Given the homeostatic roles of TGF*β* in inflammation, immune regulation, and carcinogenesis, perhaps a better strategy for TGF*β* inhibition would be to specifically target tissue-restricted activators of TGF*β* such as the *α*v*β*6 integrin. In patients with IPF and SSc ILD with a UIP pattern, the *α*v*β*6 integrin is highly upregulated on lung epithelium, implicating this pathway in TGF*β* activation [56]. In the only published report to date, upregulation of *α*v*β*6 was found on lung epithelium in seven out of seven SSc patients with UIP and in a single patient with SSc ILD who had fibrotic NSIP, but not in patients with cellular NSIP, however, the numbers of patients with NSIP analyzed were too small to draw meaningful conclusions [56]. It would therefore be important to better characterize whether upregulation of *α*v*β*6 specifically segregates with the UIP and fibrotic NSIP subsets of SSc ILD, and what role, if any, this integrin plays in the cellular NSIP subset. Anecdotal evidence and case series suggest that immunomodulators might more effectively target the cellular NSIP subset of SSc ILD, whereas the fibrotic NSIP and UIP subsets are thought to be more recalcitrant to currently available therapies [58]. Of particular interest, a mouse model of radiation-induced lung fibrosis identified a sharp upregulation of *α*v*β*6 expression by immunohistochemical analysis at 18 weeks following radiation challenge, with staining seen only in regions of fibrosis [46] and similar upregulation in fibrotic regions was found in lungs of IPF patients [56]. It thus appears that the induction of *α*v*β*6 correlates closely with fibrosis and that this integrin is often present at high concentrations in regions where active TGF*β* could be contributing to disease progression.

A highly potent-blocking antibody to the *α*v*β*6 integrin was developed and shown to prevent fibrosis in mouse models of bleomycin- and radiation-induced lung fibrosis [46, 56]. In these studies, near maximal effects on collagen production were obtained at 1 mg/kg weekly dosing of the antibody. Importantly, a treatment (as opposed to prophylaxis) trial was performed in mice by giving the *α*v*β*6-blocking antibody at day 15 following intratracheal bleomycin administration, and decreased fibrosis at day 60 was observed using the hydroxyproline assay to measure lung collagen content. Given the finding of low-grade inflammation in the lungs of the *β*6 deficient mice [43] as well as their late stage development of emphysema, a process that was dependent on MMP12 [44], a concerted effort was made to characterize whether a similar inflammatory phenotype with elevated MMP12 levels was observed in mice receiving the *α*v*β*6-blocking antibody. Transcript profiling of the lungs of mice treated with high doses (10 mg/kg) of the *α*v*β*6 blocking antibody paralleled the changes seen in *β*6 integrin knockout mice, including upregulation of MMP12 levels. Importantly, at lower doses of the *α*v*β*6 blocking antibody (1 mg/kg or 3 mg/kg), MMP12 induction was greatly diminished [56], and BAL cell counts and inflammatory cytokines were not different than in saline-treated mice [46, 56]. At these lower doses of blocking antibody, significant inhibition of collagen production was still observed, as assessed by an *in vivo* collagen luciferase reporter system, suggesting that the antifibrotic effect of *α*v*β*6 inhibition could be uncoupled from the proinflammatory effect. Induction of TGF*β* activation by bleomycin, as measured by phospho-Smad levels in lung lysates, was completely blocked at the 3 mg/kg but not by the 1 mg/kg dose of *α*v*β*6 blocking antibody suggesting that complete blockade of TGF*β* signaling is not required to achieve antifibrotic efficacy and inhibition of TGF*β*-induced fibrosis can be achieved without excessively perturbing the homeostatic functions of TGF*β*. 

Treatment of healthy, unchallenged mice with high doses of the *α*v*β*6 blocking antibody has been shown to lead to mixed cellular infiltrates (macrophages, lymphocytes, neutrophils) in lung tissue, not dissimilar to the inflammation seen in the *β*6 knockout mice. However, long-term treatment of healthy primates with a humanized form of the same *α*v*β*6 blocking antibody leads to a minimal to mild increase in lung macrophages, which resolves completely following discontinuation of treatment, with no increase in mixed cellular inflammation (unpublished observations). These findings have suggested that inhibition of *α*v*β*6 does not induce the same degree of inflammation in primates as seen in mice. Additionally, no evidence of emphysema has been observed after 6 months of weekly dosing with high doses of *α*v*β*6 antibody in mice or primates and there has been no evidence of elevated MMP-12 expression in primates with *α*v*β*6 antibody treatment as observed in mice.

## 6. Conclusions

Inhibition of *α*v*β*6 as a means of locally dampening TGF*β* activation by epithelial cells provides a rational therapeutic approach for conditions such as lung fibrosis. Importantly, the antifibrotic effect of *α*v*β*6 inhibition can be achieved at a dose that is uncoupled from its proinflammatory effect in mice [46, 56]. A phase II trial using a humanized *α*v*β*6 blocking antibody (STX-100) in IPF patients will soon be underway, and these results should be of considerable interest to the SSc community. Evaluation of the utility of inhibition of *α*v*β*6-mediated TGF*β* activation in SSc ILD, particularly the UIP and fibrotic NSIP subgroups, may be worth considering, especially if these early studies in IPF prove promising. In addition, recent data implicate an important role for epidermal keratinocytes in SSc skin fibrosis [59]. *α*v*β*6 is induced on injured keratinocytes in other settings, so the expression of *α*v*β*6 should be more closely evaluated in skin samples from SSc patients to determine whether a subset of these patients might also benefit from *α*v*β*6 blockade for treatment of skin fibrosis. 

Given the known heterogeneity of SSc within and beyond the limited and diffuse subsets [60, 61], the inhibition of epithelial *α*v*β*6-mediated TGF*β* activation may not address some of the other manifestations of SSc, in particular the vascular complications in which endothelial injury has been posited as an initiating mechanism. In fact, it is unlikely that any single treatment strategy will effectively combat the various pathologic manifestations of SSc. Whether the mechanisms leading to fibrosis of the skin and other internal organs in SSc are dependent upon *α*v*β*6-mediated TGF*β* activation remains to be determined. Additional mechanisms involved in TGF*β* activation, such as the integrins *α*v*β*3, *α*v*β*5, and *α*v*β*8, could be playing a contributory role, but discussion of this is beyond the scope of the current paper. 

Importantly, when considering strategies that target TGF*β* activity, potential side effects should be carefully monitored, such as the development of aberrant inflammation or cancer. However, in light of the morbidity and mortality associated with fibrotic lung diseases, especially IPF or the more fibrotic phenotypes of SSc ILD (UIP and fibrotic NSIP), perhaps these treatment risks can be justified given the lack of alternatives short of lung transplantation in some cases. TGF*β* activity seems to be the “Achilles heel” of pulmonary fibrosis, and the ability to locally inhibit its activity presents an attractive strategy that may likely be met with clinical success.
